# His-Purkinje system pacing versus biventricular pacing in clinical efficacy: a systematic review and meta-analysis

**DOI:** 10.1186/s12872-023-03307-7

**Published:** 2023-06-03

**Authors:** Ya Wang, Fangchao Liu, Mengyao Liu, Zefeng Wang, Xiangfeng Lu, Jianfeng Huang, Dongfeng Gu

**Affiliations:** 1 Department of Epidemiology, Fuwai Hospital, State Key Laboratory of Cardiovascular Disease, National Center for Cardiovascular Diseases, Chinese Academy of Medical Sciences and Peking Union Medical College, No. 167 Beilishi Road, Beijing, 100037 China; 2 Department of Cardiology, Beijing Anzhen Hospital Affiliated to Capital Medical University, Beijing, China; 3 School of Medicine, Southern University of Science and Technology, Shenzhen, 518055 China

**Keywords:** His-purkinje system pacing, Biventricular pacing, Cardiac resynchronization therapy, Meta-analysis

## Abstract

**Background:**

His-Purkinje system pacing (HPSP), including his-bundle pacing (HBP) and left bundle branch area pacing (LBBaP), imitates the natural conduction of the heart as an alternative to biventricular pacing (BVP) in cardiac resynchronization therapy (CRT). However, the feasibility and efficacy of HPSP were currently only evidenced by studies with a limited sample size, so this study aimed to provide a comprehensive assessment through a systematic review and meta-analysis.

**Methods:**

In order to compare the clinical outcomes associated with HPSP and BVP in patients for CRT, PubMed, EMBASE, Cochrane Library and Web of Science database were searched from inception to April 10, 2023. Clinical outcomes of interest including QRS duration (QRSd), left ventricular (LV) function and New York Heart Association (NYHA) functional classification, pacing threshold, echocardiographic and clinical response, hospitalization rate of HF and all-cause mortality were also extracted and summarized for meta-analysis.

**Results:**

A total of 13 studies (ten observational studies and three randomized studies) involving 1,121 patients were finally included. The patients were followed up for 6–27 months. Compared with BVP, CRT patients treated by HPSP presented shorter QRSd [mean difference (MD): -26.23 ms, 95% confidence interval (CI): -34.54 to -17.92, *P* < 0.001, *I*^*2*^ = 91%], greater LV functional improvement with increased left ventricular ejection fraction (LVEF) (MD: 6.01, 95% CI: 4.81 to 7.22, *P* < 0.001, *I*^*2*^ = 0%), decreased left ventricular end-diastolic dimension (LVEDD) (MD: -2.91, 95% CI: -4.86 to -0.95, *P* = 0.004, *I*^*2*^ = 35%), and more improved NYHA functional classification (MD: -0.45, 95% CI: -0.67 to -0.23, *P* < 0.001, *I*^*2*^ = 70%). In addition, HPSP was more likely to have higher echocardiographic [odds ratio (OR): 2.76, 95% CI: 1.74 to 4.39, *P* < 0.001,* I*^*2*^ = 0%], clinical (OR: 2.10, 95% CI: 1.16 to 3.80, *P* = 0.01, *I*^*2*^ = 0%) and super clinical (OR: 3.17, 95% CI: 2.09 to 4.79, *P* < 0.001, *I*^*2*^ = 0%) responses than BVP, and a lower hospitalization rate of HF (OR: 0.34, 95% CI: 0.22 to 0.51, *P* < 0.001, *I*^*2*^ = 0%), while presented no difference (OR: 0.68, 95% CI: 0.44 to 1.06, *P* = 0.09, *I*^*2*^ = 0%) in all-cause mortality compared with BVP. With threshold change taking into account, BVP was less stable than LBBaP (MD: -0.12 V, 95% CI: -0.22 to -0.03, *P* = 0.01, *I*^*2*^ = 57%), but had no difference with HBP (MD: 0.11 V, 95% CI: -0.09 to 0.31, *P* = 0.28, *I*^*2*^ = 0%).

**Conclusion:**

The present findings suggested that HPSP was associated with greater improvement of cardiac function in patients with indication for CRT and was a potential alternative to BVP to achieve physiological pacing through native his-purkinje system.

**Graphical Abstract:**

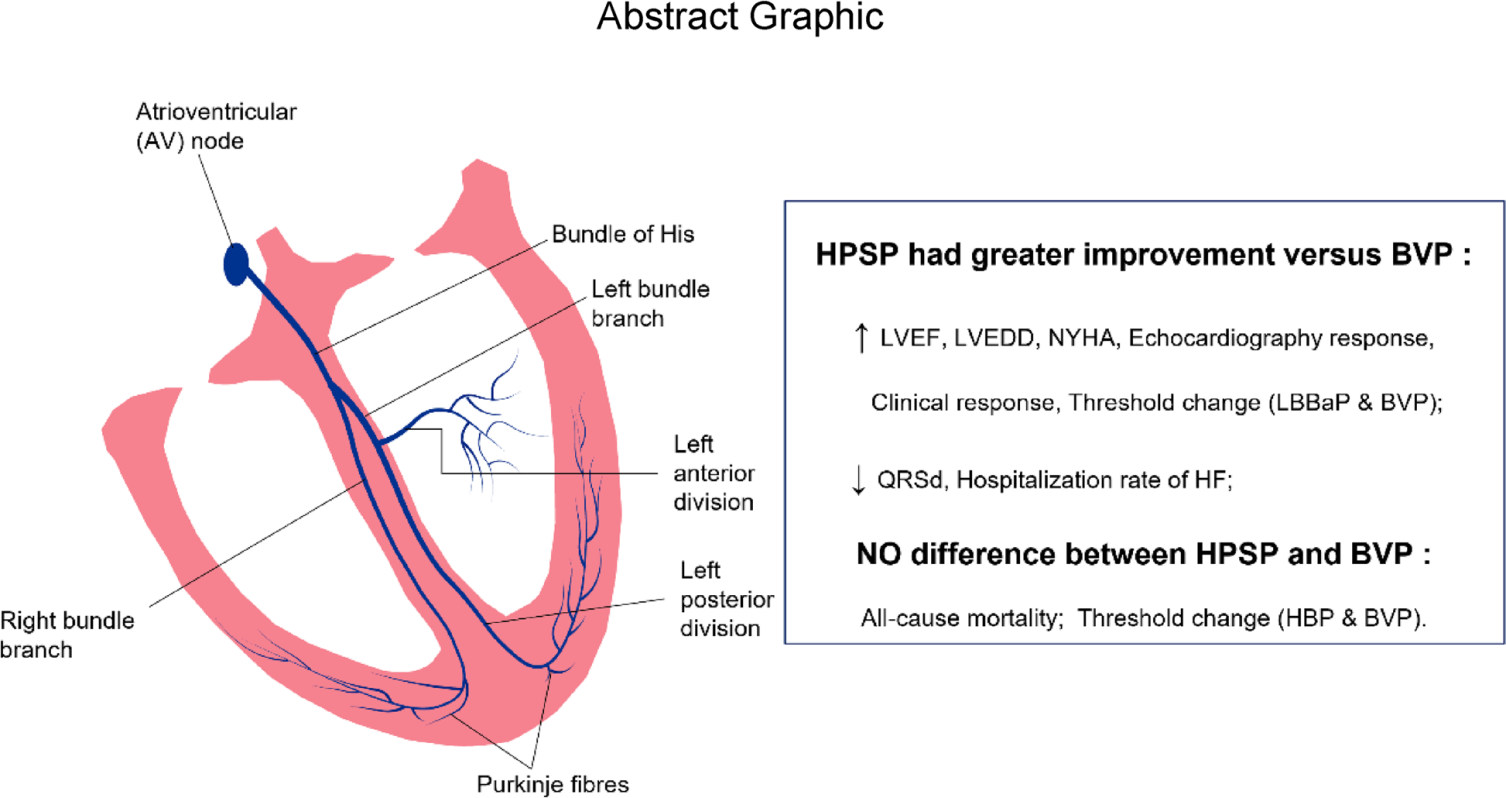

**Supplementary Information:**

The online version contains supplementary material available at 10.1186/s12872-023-03307-7.

## Introduction

Cardiac resynchronization therapy (CRT) can effectively improve the symptoms of patients with heart failure (HF) and cardiac electromechanical dyssynchrony. A large number of randomized clinical trials have demonstrated the efficiency of biventricular pacing (BVP) in improving left ventricular (LV) function as well as New York Heart Association (NYHA) functional classification, and reducing rehospitalization in patients with HF and cardiac conduction block [1–6]. However, 30% ~ 40% of patients having received BVP therapy have no response to CRT due to some limitations [7–10]. Besides, it is well recognized that BVP is not a true physiological electromechanical synchronization through fusing LV epicardial wavefront with endocardial wavefront of the right ventricular apex and native cardiac conduction. Apart from that, BVP implantation is limited by coronary venous anatomy [11, 12].

Subsequently, new physiological pacing method, i.e., his-purkinje system pacing (HPSP), was developed gradually, which included his-bundle pacing (HBP) and left bundle branch pacing area (LBBaP). In 2000, Deshmukh et al. first reported the HBP activated intrinsic conduction system through the his-purkinje system [13]. Then, Lustgarten et al. demonstrated the similar clinical outcomes of HBP compared with BVP [14]. Currently, some clinical trials recommended HBP as an alternative and frontline therapy for patients with CRT-indication [15–17]. In 2017, LBBaP, which delivered physiological pacing via capturing the LV conduction system, was first reported by Huang et al., who observed narrowed QRS complexes and improved LV function for LBBaP in HF patients [18]. Additionally, left bundle branch blocks (LBBB) can be rectified by LBBaP and the cardiac function can effectively be improved [19].

BVP still remains first-line therapy in view of the substantial evidence of efficacy and safety. However, HPSP is very likely to play a growingly important role in the future, and the current recommendations may need to be revised if future long-term evidence of safety and efficacy is published [20]. To this end, this meta-analysis was hereby conducted to evaluate clinical outcomes of HPSP versus BVP on cardiac synchronization.

## Methods

This meta-analysis was conducted in accordance with the Preferred Reporting Items for Systematic Reviews and Meta-Analyses (PRISMA) [21] during the whole process.

### Search strategy

Related articles published from inception to April 10, 2023 were selected, and PubMed, EMBASE, Cochrane’s Library and Web of Science were searched using the following terms: (1) “left bundle branch area pacing” and “biventricular” OR “cardiac resynchronization therapy” OR “CRT”; (2) “His-bundle pacing” and “biventricular” OR “cardiac resynchronization therapy” OR “CRT”; and (3) “conduction system pacing” and “biventricular” OR “cardiac resynchronization therapy” OR “CRT”. Details on search strategies are shown in Supplementary materials. There is no restriction on language and study. In addition, the references of the retrieved studies were also manually screened for relevant potential studies.

### Selection criteria and study selection

Case report, abstract, review, editorial, letter or study involving less than ten patients were excluded. Inclusion criteria were as follows: (1) randomized or non-randomized controlled trial (RCT) and observational study; (2) studies comparing LBBaP or HBP with BVP among patients with HF; and (3) studies investigating one of the following outcomes: QRS duration (QRSd), echocardiographic parameters [left ventricular ejection fraction (LVEF) and/or left ventricular end-diastolic dimension (LVEDD)], NYHA functional classification, echocardiographic response, clinical and super clinical CRT response rate, pacing threshold, hospitalization rate of HF and all-cause mortality.

Echocardiographic response was defined as an LVEF improvement of at least 5% at the end of the follow-up, and clinical response was defined as an improvement in the patient symptom, such as decreased NYHA functional classification of at least one grade at the last follow-up [22], while super response was defined as a remarkable improvement in cardiac function: NYHA functional classification decreased to I or II, along with an improvement in LVEF of at least 15% or eventual LVEF more than 45% [23].

Two investigators independently screened the titles and abstracts, full text of potential eligible articles according to the inclusion criteria. Any disagreement was resolved by discussing with the third investigator.

### Data extraction

Two independent investigators conducted the data extraction from the included studies, and disagreements were resolved by consensus and discussions with the third investigator. The following information was extracted from studies: general study information (including first author, country and year of publication), study characteristics (including study design, article type, sample size and follow-up), participant characteristics (including age, sex and indication of implantation) and efficacy indexes [QRSd, echocardiographic parameters (LVEF and LVEDD), NYHA functional classification, pacing threshold, echocardiographic response rate, clinical and super clinical CRT response rate, hospitalization rate of HF, all-cause mortality].

### Quality evaluation

Two independent investigators assessed the quality of the included RCT with the Cochrane’s Risk of Bias Tool [24]. Quality of observational studies was evaluated with the Newcastle–Ottawa Scale (NOS) [25]. NOS score of greater than or equal to 6 points was defined as high-quality, while that less than 6 points was considered low-quality.

### Data synthesis and statistical analyses

Mean difference (MD) of the difference between the last follow-up and baseline was used to measure the outcomes of continuous variables, while odds ratio (OR) was used for the categorized variables. Once the variance for the paired difference before and after the intervention was not reported, the equation of (*σ*^2^_▲_ = *σ*^2^_pre_ + *σ*^2^_post_—2*ρσ*_*pre*_*σ*_*post*_) was used [26], where, σ_pre_ represents the variance at baseline; σ_post_, the variance at the end of the follow-up; and ρ, the correlation coefficient for correlations before and after the intervention. In accordance with practice, this analysis was conducted with an estimated ρ value of 0.5.

A random-effect model was used to generate pooled estimates of the difference, and heterogeneity was assessed by the *Q* and *I*^*2*^ statistics. In the presence of heterogeneity, subgroup analysis was performed to find the possible source. Meanwhile, sensitivity analyses were performed to assess the robustness of the finding: except for implementing inclusion and exclusion criteria strictly, each trial was also excluded sequentially from the meta-analysis to determine the influence of a single study, and potential publication bias was assessed using funnel plots.

Review Manager (RevMan Version 5.4, The Cochrane Collaboration, 2020, Oxford, United Kingdom) and R software (version 4.1.2) were used for the statistical analysis. A 2-sided *P* value of < 0.05 was considered statistically significant.

## Results

### Study selection

**Fig. 1:**
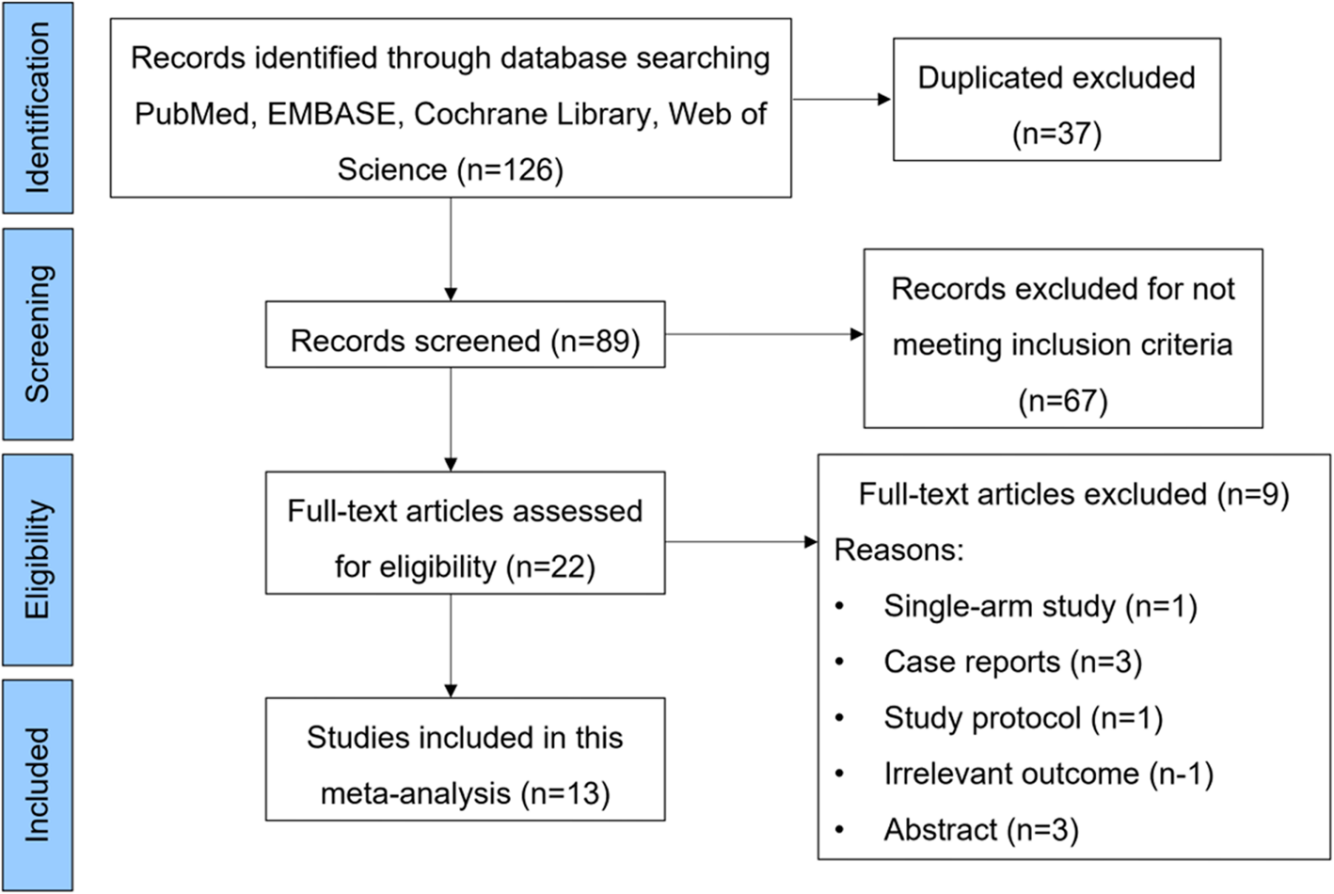
Flowchart of database search and study identification

Initially, a total of 126 potentially eligible articles were retrieved from the databases (Fig. 1), and 89 records were left after exclusion of duplications. Of these, by screening via the titles and abstracts, 67 were subsequently excluded, and 13 [14, 27–38] were included in this meta-analysis after further excluding single-arm studies, study protocols, and studies with irrelevant outcomes.

### Study characteristics and quality evaluation

**Fig. 2:**
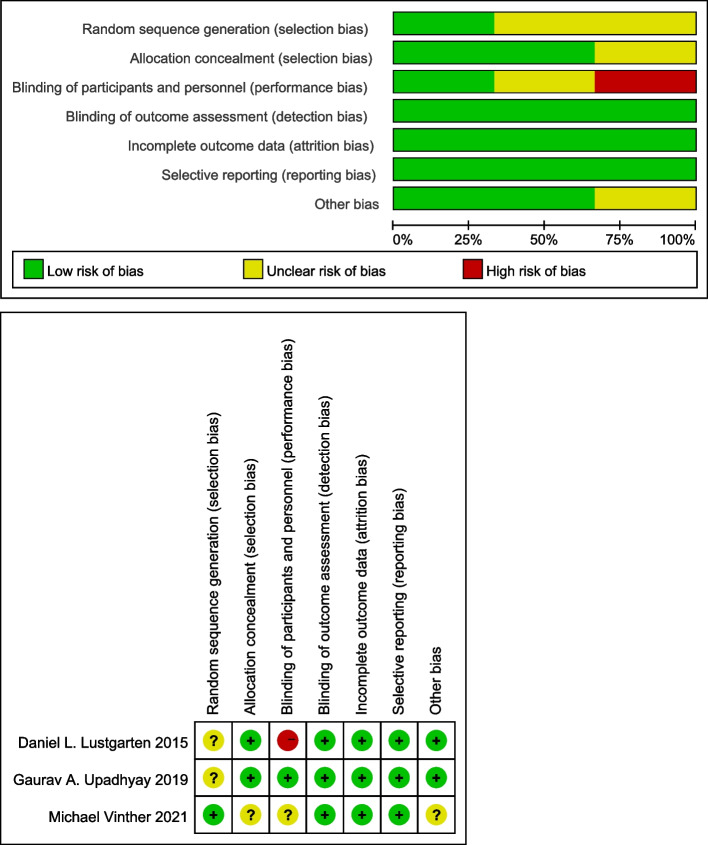
Quality judgements about each risk of bias item presented as percentages across three RCT studies

Ten of the included articles [27–31, 34–38] were observational studies, and three [14, 33] were randomized studies. The meta-analysis included 1,121 patients recruited from 29 centers in all. Patients who received HPSP (LBBaP or HBP) and BVP were generally frequency-matched on age and sex (Table 1), period of the follow up were six months in seven studies [14, 27–29, 32, 33, 36], 12 months in two studies [30, 35], 14 months [34], 18 months [31], 24 months [37] and 27 months [38] in one study each. All patients had HF and CRT indication in 13 studies [14, 27–38]. In 9 studies [27–30, 32, 34–38], all of the patients (68%, *n* = 763) had LBBB, while in 8 studies [28–32, 34, 35, 38], the majority of the subjects had non-ischemic cardiomyopathy (63%, *n* = 584). Meanwhile, 92% (*n* = 972) of the population used β-blockers in 11 studies [27–30, 32–38], and 76.4% (*n* = 847) used Angiotensin-Converting Enzyme Inhibitors (ACEI)/ Angiotensin Receptor Blockers (ARB)/ Angiotensin Receptor-Neprilysin Inhibitor (ARNI) in 12 studies [27–38]. All included [14, 27–38] studies were graded with a score of greater than 6 points, and none of the included studies was of poor quality. Quality assessment of the included studies is presented in Table 2 and Fig. 2.

**Table 1:** Characteristics of the included studies

**Author**	**Country**	**Year**	**Article type**	**Design**	**Indication**	**Age**		**Sample size**		**Male(%)**		**Follow-up(M)**
						**LBBaP/HBP**	**BVP**	**LBBaP/HBP**	**BVP**	**LBBaP/HBP**	**BVP**	
Lustgarten et al.	U. S	2015	Randomized trial	HBP vs BVP	QRS > 130 ms	71.3 (55.91–86.75)	71.3 (55.91–86.75)	12	12	66.7	66.7	6
Upadhyay et al.	U. S	2019	Randomized trial	HBP vs BVP	NYHA II-IV with QRS > 120 ms	63.4 ± 13.3	65.5 ± 12.4	16	24	56.2	66.7	6
Vijayaraman et al.	U. S	2019	Observational study	HBP vs BVP	NYHA III-IV, LVEF ≤ 35%, LBBB and IVCD with QRS ≥ 140 ms	72 ± 15	72 ± 15	27	27	85	85	14
Li et al.	China	2020	Observational study	LBBaP vs BVP	HF, LVEF ≤ 35%, LBBB	57.5 ± 9.8	58.5 ± 8.5	27	54	51.9	61.1	6
Guo et al.	China	2020	Observational study	LBBaP vs BVP	HF, LVEF ≤ 35%, LBBB, NYHA II-IV	66.1 ± 9.7	65.1 ± 7.5	21	21	42.9	42.9	6
Wang et al.	China	2020	Observational study	LBBaP vs BVP	HF, CLBBB with QRS > 130 ms, LVEF ≤ 35%, NYHA II-IV	64.8 ± 7.25	62.9 ± 10.33	10	30	90	76.6	6
Chen et al.	China	2021	Observational study	LBBaP vs BVP	HF, LBBB with QRS > 150 ms, LVEF ≤ 35%, NYHA II-IV	67.14 ± 8.88	64.37 ± 8.74	49	51	49.98	58.82	12
Wu et al.	China	2021	Observational study	LBBaP or HBP vs BVP	HF, LVEF ≤ 40%, CLBBB	67.2 ± 13/68.3 ± 10	68.3 ± 10	32/49	54	43.8/63.3	53.7	12
Ma et al.	China	2021	Observational study	HBP vs BVP	Brady-arrhythmia, Permanent AF and HF with HFrEF	70.32 ± 12.36	66.8 ± 7.26	37	15	70.27	66.7	18
Vinther et al.	Denmark	2021	Randomized trial	HBP vs BVP	HF with LVEF ≤ 35%, NYHA II-IV, LBBB with QRS > 130 ms	63.2 ± 9.2	67.4 ± 9.1	19	31	42	77	6
Zizek et al.	Slovenia	2021	Observational study	HBP vs BVP	Permanent AF, 35% ≤ LVEF < 50%, QRS ≤ 120 ms,NYHA II-III	68.5 ± 6.8	69.3 ± 6.6	12	12	41.7	58.3	6
Hua et al.	China	2022	Observational study	LBBaP vs BVP	HF with NYHA II-IV, QRS ≥ 150, CLBBB	65.5 ± 6.91	67.5 ± 11.69	21	20	15	16	24
Vijayaraman et al.	U. S	2022	Observational study	CSP vs BVP	LVEF ≤ 35% with class II-IV heart failure symptoms	72 ± 13	72 ± 12	258	219	170	156	27

*IVCD* intraventricular conduction delay, *CLBBB* complete left bundle branch block, *HFrEF* heart failure with reduced ejection fraction, *AF* atrial fibrillation, *CSP *LBBaP and HBP

**Table 2:** Details of study quality evaluation via the NOS

Study	Representativeness of the patient	Selection of the controls	Ascertainment of intervention	Demonstration that outcome of interest was not present at the start of the study	Comparability age and gender	Comparability other factors	Assessment of outcome	Was follow-up long enough for outcomes to occur	Adequacy of follow-up of cohorts	Total
Li	1	1	1	1	1	1	1	0	1	8
Guo	1	1	1	1	1	1	1	0	1	8
Wang	1	1	1	1	1	1	1	0	1	8
Wu	1	1	1	1	1	1	1	1	1	9
Ma	1	1	1	1	1	1	1	1	1	9
Zizek	1	1	1	1	1	1	1	0	1	8
Vijayaraman	1	1	1	1	1	1	1	1	1	9
Hua	1	1	1	1	1	1	1	1	1	9
Chen	1	1	1	1	1	1	1	1	1	9
Vijayaraman	1	1	1	1	1	1	1	1	1	9

### Change of QRSd

**Fig. 3:**
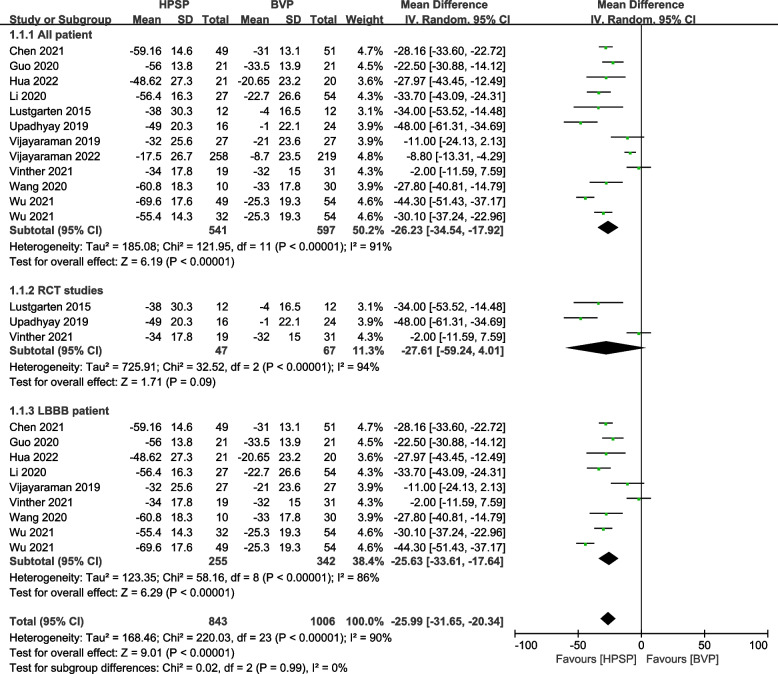
Forest plot of mean difference (MD) of cardiac resynchronization therapy (CRT) on QRS duration (QRSd) decrease in different groups. Net changes in QRSd were estimated by comparing BVP with HPSP. Squares indicate the effect size of a study, with a 95% CI; and diamonds indicate the overall effect size of all studies combined

Articles with wide QRSd were analyzed using a random-effect model in different groups. The result of 11 studies [14, 27–30, 32–35, 37, 38] showed that the HPSP group had a significantly greater reduction in a mean of QRSd compared with BVP (MD: -26.25 ms, 95% confidence interval (CI): -34.54 to -17.92, *P* < 0.001, *I*^*2*^ = 91%). Similarly, in patients with LBBB, BVP presented a less reduction in a mean of QRSd than HPSP (MD: -25.63 ms, 95% CI: -33.61 to -17.64, *P* < 0.001, *I*^*2*^ = 86%) in 8 studies [27–30, 32, 34, 35, 37]. No difference in a mean of QRSd was observed between HPSP and BVP in three RCT studies [14, 32, 33] (MD: -27.61 ms, 95% CI: -59.24 to 4.01, *P* < 0.001, *I*^*2*^ = 94%) (Fig. 3).

### LV function and NYHA functional classification assessment

**Fig. 4:**
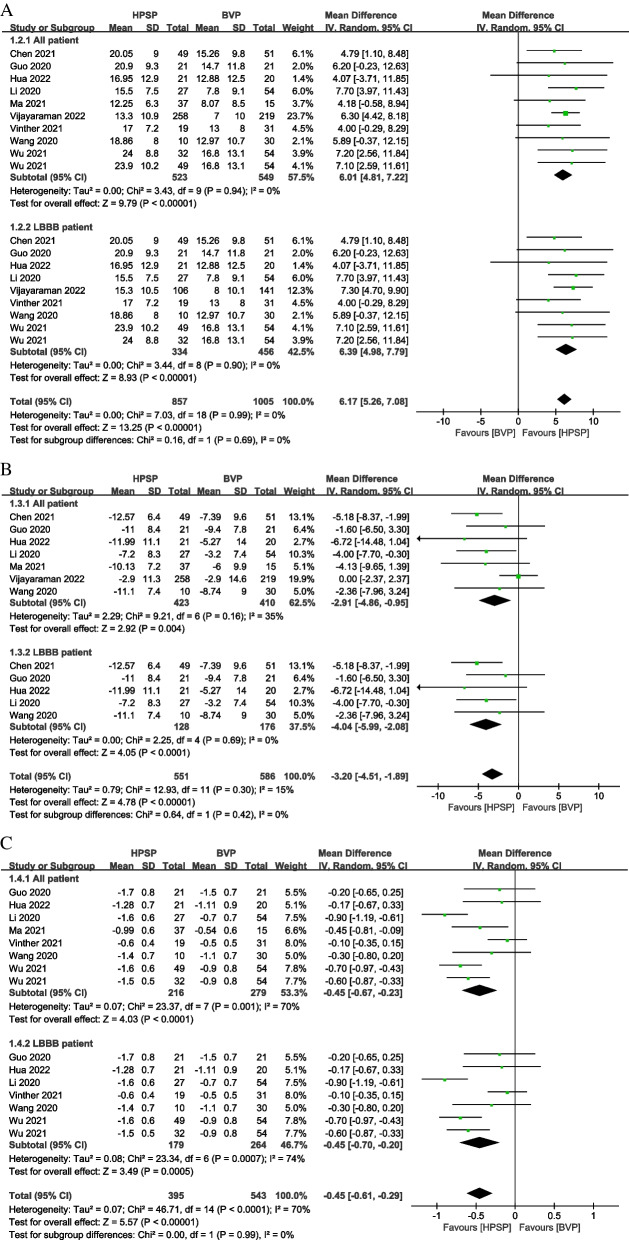
Forest plot of the effect of cardiac resynchronization therapy (CRT) on left ventricular function and New York Heart Association (NYHA) functional classification improvement in different groups. Net changes in left ventricular ejection fraction (LVEF) (**A**); left ventricular end-diastolic dimension (LVEDD) (**B**); and NYHA functional classification (**C**) were estimated by comparing HPSP with BVP. Squares indicate the effect size of a study, with a 95% CI; and diamonds indicate the overall effect size of all studies combined

Pooled analysis using a random-effect model from 9 studies [27–32, 35, 37, 38] suggested that compared with BVP, HPSP was associated with a significant improvement in LVEF (MD: 6.01, 95% CI: 4.81 to 7.22, *P* < 0.001, *I*^*2*^ = 0%). Consistently, LVEF in HPSP improved to a greater degree (MD: 6.39, 95% CI: 4.98 to 7.79, *P* < 0.001, *I*^*2*^ = 0%) than BVP in LBBB patients from 8 studies [27–30, 32, 35, 37, 38] (Fig. 4A). LVEDD from 7 articles [27–29, 31, 35, 37, 38] decreased significantly in HPSP compared with BVP (MD: -2.91, 95% CI: -4.86 to -0.95, *P* = 0.004, *I*^*2*^ = 35%). Among patients with LBBB from 5 studies [27–29, 35, 37], LVEDD in HPSP also presented a significant decrease compared with BVP (MD: -4.04, 95% CI: -5.99 to -2.08, *P* < 0.001, *I*^*2*^ = 0%) (Fig. 4B). The difference in NYHA functional classification improvement was assessed in 7 studies [27–32, 37], and the results indicated that compared with BVP, HPSP had more improvement in NYHA functional classification (MD: -0.45, 95% CI: -0.67 to -0.23, *P* < 0.001, *I*^*2*^ = 70%). In patients with LBBB, HPSP also improved significantly compared with BVP (MD: -0.45, 95% CI: -0.70 to -0.20, *P* = 0.0005, *I*^*2*^ = 74%) in 6 studies [27–30, 32, 37] (Fig. 4C).

### Threshold change

**Fig. 5:**
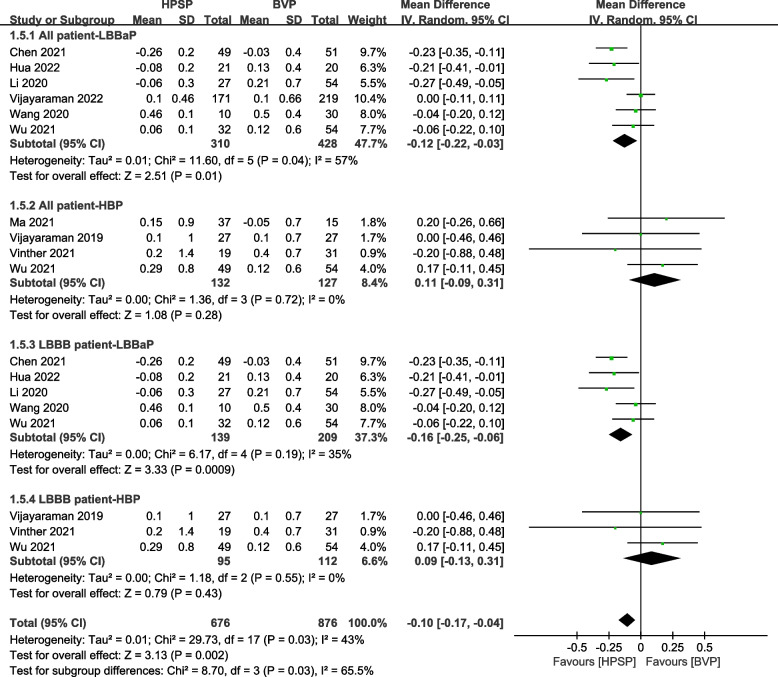
Forest plot of the effect of cardiac resynchronization therapy (CRT) on threshold change in different groups. Net changes in threshold change were estimated by comparing BVP with LBBaP and HBP

The difference in threshold change was assessed using the random-effect model in 6 studies [27, 29, 30, 35, 37, 38] in LBBaP and 4 studies [30–32, 34] in HBP, and it was found that the performance was more stable in LBBaP than in BVP (MD: -0.12 V, 95% CI: -0.22 to -0.03, *P* = 0.01, *I*^*2*^ = 57%), while no significant difference was observed between HBP and BVP (MD: 0.11 V, 95% CI: -0.09 to 0.31, *P* = 0.28, *I*^*2*^ = 0%). Similarly, in LBBB patients, BVP had a greater change (MD: -0.16 V, 95% CI: -0.25 to -0.06, *P* = 0.0009, *I*^*2*^ = 35%) than LBBaP in 5 studies [27, 29, 30, 35, 37], and no significant difference was observed among HBP and BVP patients (MD: -0.09 V, 95% CI: -0.13 to 0.31, *P* = 0.43, *I*^*2*^ = 0%) in 3 studies [30, 32, 34] (Fig. 5).

### CRT response rate

**Fig. 6:**
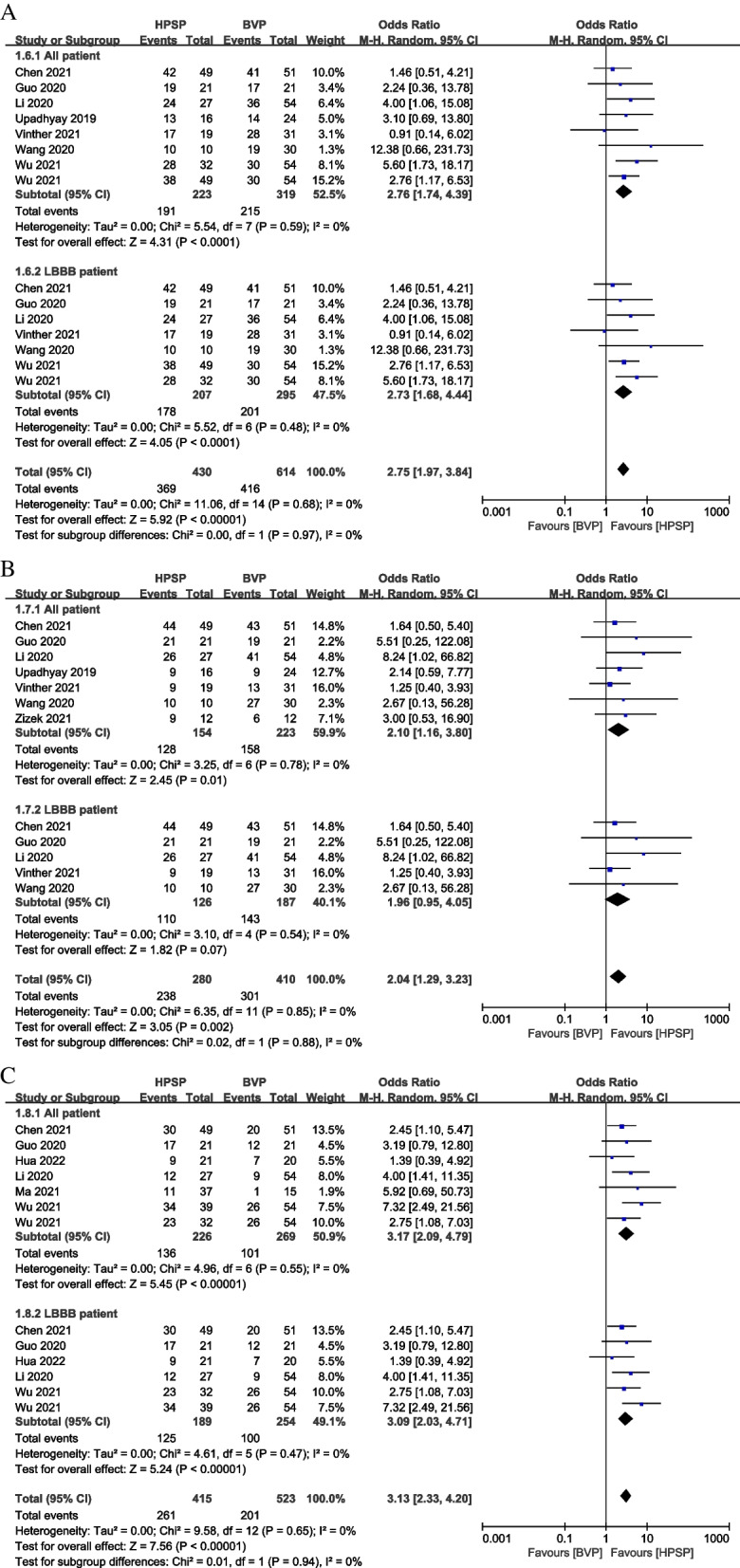
Forest plot of odds ratio (OR) on cardiac resynchronization therapy (CRT) response in different groups. echocardiographic response (**A**); clinical response (**B**); and super clinical response (**C**) were estimated by comparing HPSP with BVP. Squares indicate the effect size of a study, with a 95% CI; and diamonds indicate the overall effect size of all studies combined

Pooled analysis was carried out using the random-effect model, and the results suggested that compared with BVP, HPSP patients were likely to achieve higher (OR: 2.76, 95% CI: 1.74 to 4.39, *P* < 0.001, *I*^*2*^ = 0%) echocardiographic response in 7 studies [27–30, 32, 33, 35]. In LBBB patients, BVP had lower (OR: 2.73, 95% CI: 1.68 to 4.44, *P* < 0.001, *I*^*2*^ = 0%) echocardiographic response than HPSP in 6 studies [27–30, 32, 35] (Fig. 6A). Meanwhile, in 7 studies [27–29, 32, 33, 35, 36], HPSP had a higher clinical (OR: 2.10, 95% CI: 1.16 to 3.80, *P* = 0.01, *I*^*2*^ = 0%) response than BVP, and there was no difference (OR: 1.96, 95% CI: 0.95 to 4.05, *P* = 0.07, *I*^*2*^ = 0%) between HPSP and BVP in LBBB patients from 5 studies [27–29, 32, 35] (Fig. 6B). HPSP patients had a significantly super clinical (OR: 3.17, 95% CI: 2.09 to 4.79, *P* < 0.001, *I*^*2*^ = 0%) response compared with BVP in 6 studies [27, 28, 30, 31, 35, 37]. Among patients with LBBB, BVP had a lower super clinical response (OR: 3.09, 95% CI: 2.03 to 4.71, *P* < 0.001, *I*^*2*^ = 0%) than HPSP in 5 studies [27, 28, 30, 35, 37] (Fig. 6C).

### Hospitalization rate of HF and all-cause mortality

**Fig. 7:**
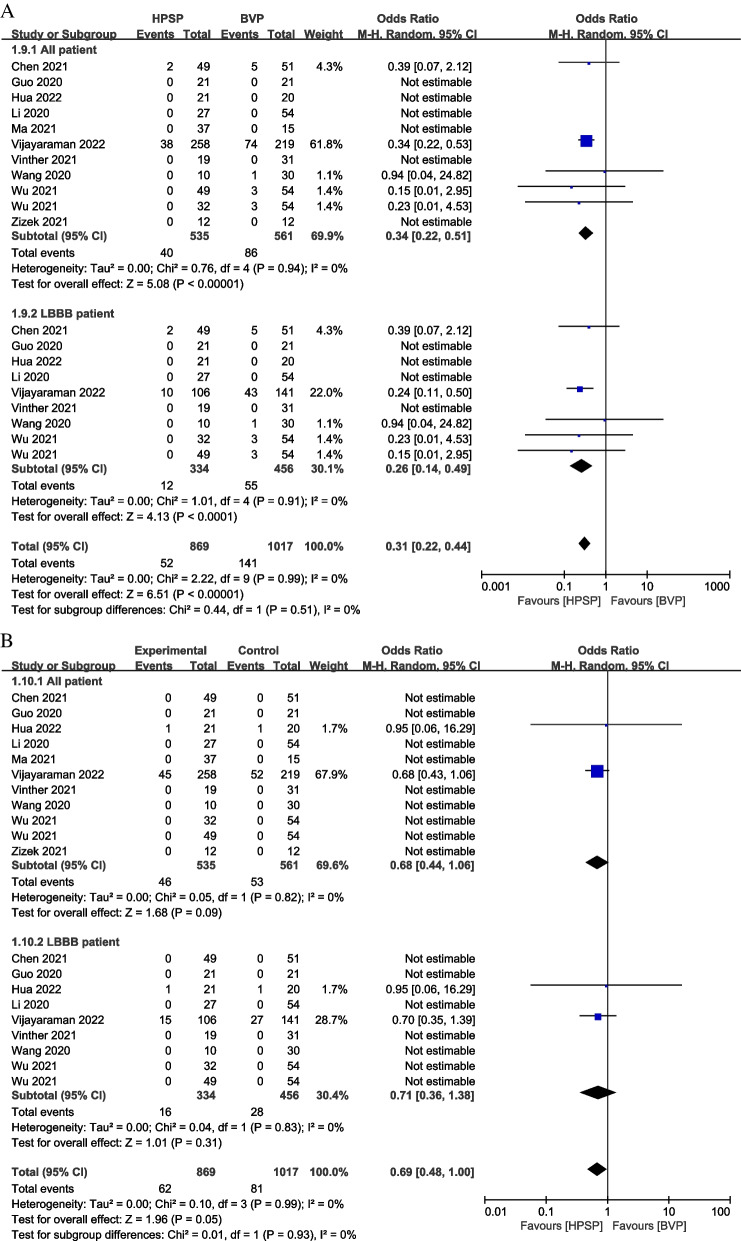
Forest plot of odds ratio (OR) on rehospitalization and all-cause mortality in different groups. The hospitalization rate of HF (**A**) and all-cause mortality clinical response (**B**) were estimated by comparing HPSP with BVP. Squares indicate the effect size of a study, with a 95% CI; and diamonds indicate the overall effect size of all studies combined

The hospitalization rate of HF was higher in BVP than HPSP in 10 studies (OR: 0.34, 95% CI: 0.22 to 1.06, *P* < 0.001, *I*^*2*^ = 0%) [27–32, 35–38] and patients with LBBB in 8 studies (OR: 0.26, 95% CI: 0.14 to 0.49, *P* < 0.001, *I*^*2*^ = 0%) [27–30, 32, 35, 37, 38] (Fig. 7A). Besides, there was no statistical difference between HPSP and BVP regarding to all-cause mortality in 10 studies (OR: 0.68, 95% CI: 0.44 to 1.06, *P* = 0.09, *I*^*2*^ = 0%) [27–32, 35–38] and LBBB patients from 8 studies (OR: 0.71, 95% CI: 0.36 to 1.38, *P* = 0.31, *I*^*2*^ = 0%) [27–30, 32, 35, 37, 38] (Fig. 7B).

### Sensitivity analyses and publication bias

Sensitivity analyses were conducted by systematically excluding one study at a time, and the direction and magnitude in the hospitalization rate of HF were affected by one study [38]. In addition, there was no publication bias by visual assessment of the funnel plots in the selected outcomes of QRSd, LVEF, NYHA and threshold change (Figure S1).

## Discussion

The main findings from the present meta-analysis were as follows: Compared with BVP, HPSP provided a statistically significant decrease of QRSd, improvement of the LV function and NYHA functional classification, higher echocardiographic and clinical response, and lower hospitalization of HF. In terms of all-cause mortality, there was no difference between HPSP and BVP. Besides, LBBaP presented less threshold change than BVP, and there was no difference between HPSP and BVP in terms of threshold change.

The current analysis was advantageous in that the above clinical outcomes between HPSP and BVP were first comprehensively compared, and a further understanding of physiologic pacing was provided. Another advantage was that patients with LBBB were also observed. In this subgroup, similar results except for clinical response were obtained. After excluding one of the studies by Vinther et al. [32], there was a statistical between HPSP and BVP in clinical response, which might be attributed to the fact that BVP had a higher clinical response than HPSP in this article, while the result was opposite in other 4 studies [27–29, 35].

Prolongation of QRSd is a marker of cardiac dyssynchrony which reflects left-sided intraventricular conduction delay [39]. Decreased QRSd means a better electromechanical synchronization which can improve ventricular system function and clinical symptoms of patient [40]. The above theory was confirmed by the outcomes of the present meta-analysis once again. A subgroup composed of RCT was also added in this analysis, and the result suggested no difference between HPSP and BVP. The possible reasons were as follows: (1) the subgroup had a small sample size consisting of only three RCTs; and (2) Vinther e t al. [32], through one of the RCTs, found that His-CRT provided similar clinical and physical improvement compared with BiV-CRT.

Different areas of cardiac produce diverse pacing thresholds which are associated with the lifespan of the pacemaker. The His-bundle is a thin cylindrical fascicle connecting the AV node with the bundle branches. The penetrating portion of His-bundle is only a small distance (about 1–3 mm). Dense fibrous tissue surrounding the penetrating portion usually yields high capture threshold at implantation, and unpredictable rise in threshold may lead to premature battery depletion and lead revision. Meanwhile, LBB area is a thick band like structure that contains abundant left bundle branch (LBB) and Purkinje fibers, and electrical signals conduct across the LV rapidly. Therefore, LBBaP is provided with a unique advantage of low threshold. In the present research, LBBaP had a slower increase of threshold than HBP and BVP, and the battery of LBBaP could theoretically last longer [41]. Besides, easier implantation procedure could reduce radiation hazard. Regarding to threshold change in BVP, data of LV lead positioned in the epicardial vein were chosen for the comparison with HBP. The histological characteristic of venous tissue determines the poor conductivity and high pacing threshold in BVP, which may be the reason for no difference in threshold change between HBP and BVP in the present meta-analysis. In patients with AV node ablation, AV block and high right ventricular (RV) pacing burden, HBP is still considered an ideal and first-line strategy to achieve CRT as a physiological pacing producing electrical synchrony [13, 42]. Relatively, BVP fails to eliminate electrical dyssynchrony [43]. Herein, fluoroscopy time and operation cost were not analyzed because multi-center studies were included and the skill of implantation varied widely. The large single-center RCTs should be performed to evaluate the above outcomes.

A total of 10 studies [27–32, 35–38] reported exact number of the hospitalization rate of HF in this analysis. The result was unstable due to one of the included studies by Vijayaraman P et al. [38]. While excluding it, no difference was found between HPSP and BVP. The reason may be that HF hospitalization was the primary outcome and had a detailed definition, which was different from the situation of other studies. In addition, the influence of disease history and medication status on the hospitalization rate of HF patients having received CRT should also be considered to minimize the confounding bias.

In conclusion, HPSP is associated with a shorter QRSd and thus a lower electromechanical dyssynchrony compared with BVP, and there may be fewer adverse cardiac events. Nevertheless, there are still some patients who are not suitable for class I recommendations in the guideline or do not response to current CRTs. In order to benefit from CRT, patients need to be screened and stratified more carefully. Additionally, more optimized therapies should be further explored for the treatment of the above patients.

### Strengths and limitations

The studies included had good quality, and were from different countries with different clinical characteristics of the population. Another advantage of this analysis was that the differences between the baseline and the endpoint were adopted instead of only using the final value, which decreased the risk of confounding bias. The limitation of this study was that the proportion of RCT was not adequate. The heterogeneity might be explained by the small sample size, various follow-up duration and inconsistent baseline levels.

### Clinical perspectives

In conclusion, the HPSP could produce shorter QRSd, higher LVEF and improved NYHA functional classification compared with BVP. Otherwise, LBBaP had a more stable threshold than BVP. Overall, these findings suggested the potential of HPSP as a promising frontline strategy for CRT. Further high quality RCTs should be performed to evaluate the efficacy between HPSP and BVP.

## Supplementary Information

**Additional file 1.**

## Data Availability

All data generated or analysed during this study are included in this article.

## Abbreviations

HPSP: His-Purkinje system pacing
HBP: His-bundle pacing
LBBaP: Left bundle branch pacing area
BVP: Biventricular pacing
CRT: Cardiac resynchronization therapy
QRSd: QRS duration
LV: Left ventricular
NYHA: New York Heart Association
MD: Mean difference
CI: Confidence interval
LVEF: Left ventricular ejection fraction
LVEDD: Left ventricular end-diastolic dimension
OR: Odds ratio
HF: Heart failure
LBBB: Left bundle branch blocks
RCT: Randomized controlled trial
ACEI: Angiotensin-Converting Enzyme Inhibitors
ARB: Angiotensin Receptor Blockers
ARNI: Angiotensin Receptor-Neprilysin Inhibitor
AV: Atrioventricular
RV: Right ventricular
LBB: Left bundle branch
CSP: Conduction system pacing

## References

[1] Abraham WT, Fisher WG, Smith AL, Delurgio DB, Leon AR, Loh E (2002). Cardiac resynchronization in chronic heart failure. N Engl J Med.

[2] Cleland JG, Daubert JC, Erdmann E, Freemantle N, Gras D, Kappenberger L (2005). The effect of cardiac resynchronization on morbidity and mortality in heart failure. N Engl J Med.

[3] Daubert C, Gold MR, Abraham WT, Ghio S, Hassager C, Goode G (2009). Prevention of disease progression by cardiac resynchronization therapy in patients with asymptomatic or mildly symptomatic left ventricular dysfunction: insights from the European cohort of the REVERSE (Resynchronization Reverses Remodeling in Systolic Left Ventricular Dysfunction) trial. J Am Coll Cardiol.

[4] Moss AJ, Hall WJ, Cannom DS, Klein H, Brown MW, Daubert JP (2009). Cardiac-resynchronization therapy for the prevention of heart-failure events. N Engl J Med.

[5] Epstein AE, DiMarco JP, Ellenbogen KA, Estes NA, Freedman RA, Gettes LS (2008). ACC/AHA/HRS 2008 Guidelines for Device-Based Therapy of Cardiac Rhythm Abnormalities: a report of the American College of Cardiology/American Heart Association Task Force on Practice Guidelines (Writing Committee to Revise the ACC/AHA/NASPE 2002 Guideline Update for Implantation of Cardiac Pacemakers and Antiarrhythmia Devices) developed in collaboration with the American Association for Thoracic Surgery and Society of Thoracic Surgeons. J Am Coll Cardiol.

[6] Cazeau S, Leclercq C, Lavergne T, Walker S, Varma C, Linde C (2001). Effects of multisite biventricular pacing in patients with heart failure and intraventricular conduction delay. N Engl J Med.

[7] Daubert C, Behar N, Martins RP, Mabo P, Leclercq C (2017). Avoiding non-responders to cardiac resynchronization therapy: a practical guide. Eur Heart J.

[8] Kydd AC, Khan FZ, Ring L, Pugh PJ, Virdee MS, Dutka DP (2014). Development of a multiparametric score to predict left ventricular remodelling and prognosis after cardiac resynchronization therapy. Eur J Heart Fail.

[9] Thompson N, Derval N (2016). Left ventricular endocardial stimulation in patients with a poor response to cardiac resynchronization therapy: what is next?. JACC Clin Electrophysiol.

[10] Daubert JC, Saxon L, Adamson PB, Auricchio A, Berger RD, Beshai JF (2012). 2012 EHRA/HRS expert consensus statement on cardiac resynchronization therapy in heart failure: implant and follow-up recommendations and management. Heart Rhythm.

[11] Jarcho JA (2006). Biventricular pacing. N Engl J Med.

[12] Pothineni NVK, Supple GE (2020). Navigating challenging left ventricular lead placements for cardiac resynchronization therapy. J Innov Card Rhythm Manag.

[13] Deshmukh P, Casavant DA, Romanyshyn M, Anderson K (2000). Permanent, direct His-bundle pacing: a novel approach to cardiac pacing in patients with normal His-Purkinje activation. Circulation.

[14] Lustgarten DL, Crespo EM, Arkhipova-Jenkins I, Lobel R, Winget J, Koehler J (2015). His-bundle pacing versus biventricular pacing in cardiac resynchronization therapy patients: a crossover design comparison. Heart Rhythm.

[15] Sharma PS, Dandamudi G, Herweg B, Wilson D, Singh R, Naperkowski A (2018). Permanent His-bundle pacing as an alternative to biventricular pacing for cardiac resynchronization therapy: a multicenter experience. Heart Rhythm.

[16] Ajijola OA, Upadhyay GA, Macias C, Shivkumar K, Tung R (2017). Permanent His-bundle pacing for cardiac resynchronization therapy: Initial feasibility study in lieu of left ventricular lead. Heart Rhythm.

[17] Shan P, Su L, Zhou X, Wu S, Xu L, Xiao F (2018). Beneficial effects of upgrading to His bundle pacing in chronically paced patients with left ventricular ejection fraction <50. Heart Rhythm.

[18] Huang W, Su L, Wu S, Xu L, Xiao F, Zhou X (2017). A Novel Pacing Strategy With Low and Stable Output: Pacing the Left Bundle Branch Immediately Beyond the Conduction Block. Can J Cardiol.

[19] Zhang J, Wang Z, Cheng L, Zu L, Liang Z, Hang F (2019). Immediate clinical outcomes of left bundle branch area pacing vs conventional right ventricular pacing. Clin Cardiol.

[20] Glikson M, Nielsen JC, Kronborg MB, Michowitz Y, Auricchio A, Barbash IM (2021). 2021 ESC guidelines on cardiac pacing and cardiac resynchronization therapy. Eur Heart J.

[21] Moher D, Liberati A, Tetzlaff J, Altman DG (2009). Preferred reporting items for systematic reviews and meta-analyses: the PRISMA statement. BMJ.

[22] Parreira L (2018). Assessing response to cardiac resynchronization therapy: Time to settle on some definitive criteria. Rev Port Cardiol (Engl Ed).

[23] Rickard J, Kumbhani DJ, Popovic Z, Verhaert D, Manne M, Sraow D (2010). Characterization of super-response to cardiac resynchronization therapy. Heart Rhythm.

[24] Higgins JP, Altman DG, Gøtzsche PC, Jüni P, Moher D, Oxman AD (2011). The Cochrane Collaboration's tool for assessing risk of bias in randomised trials. BMJ.

[25] Stang A (2010). Critical evaluation of the Newcastle-Ottawa scale for the assessment of the quality of nonrandomized studies in meta-analyses. Eur J Epidemiol.

[26] Zhang X, Li Y, Del Gobbo LC, Rosanoff A, Wang J, Zhang W (2016). Effects of magnesium supplementation on blood pressure: a meta-analysis of randomized double-blind placebo-controlled trials. Hypertension.

[27] Li X, Qiu C, Xie R, Ma W, Wang Z, Li H (2020). Left bundle branch area pacing delivery of cardiac resynchronization therapy and comparison with biventricular pacing. ESC Heart Fail.

[28] Guo J, Li L, Xiao G, Ye T, Huang X, Meng F (2020). Remarkable response to cardiac resynchronization therapy via left bundle branch pacing in patients with true left bundle branch block. Clin Cardiol.

[29] Wang Y, Gu K, Qian Z, Hou X, Chen X, Qiu Y (2020). The efficacy of left bundle branch area pacing compared with biventricular pacing in patients with heart failure: a matched case-control study. J Cardiovasc Electrophysiol.

[30] Wu S, Su L, Vijayaraman P, Zheng R, Cai M, Xu L (2021). Left bundle branch pacing for cardiac resynchronization therapy: nonrandomized on-treatment comparison with his bundle pacing and biventricular pacing. Can J Cardiol.

[31] Ma PP, Yang YH, Dai BL, Zhang RF, Wang N, Li DN (2021). Brady-arrhythmias in patients with atrial fibrillation and heart failure of reduced ejection fraction: is his-bundle pacing superior to biventricular pacing?. Pacing Clin Electrophysiol.

[32] Vinther M, Risum N, Svendsen JH, Møgelvang R, Philbert BT (2021). A randomized trial of his pacing versus biventricular pacing in symptomatic HF patients with left bundle branch block (His-Alternative). JACC Clin Electrophysiol.

[33] Upadhyay GA, Vijayaraman P, Nayak HM, Verma N, Dandamudi G, Sharma PS (2019). On-treatment comparison between corrective His bundle pacing and biventricular pacing for cardiac resynchronization: a secondary analysis of the His-SYNC Pilot Trial. Heart Rhythm.

[34] Vijayaraman P, Herweg B, Ellenbogen KA, Gajek J (2019). His-optimized cardiac resynchronization therapy to maximize electrical resynchronization: a feasibility study. Circ Arrhythm Electrophysiol.

[35] Chen X, Ye Y, Wang Z, Jin Q, Qiu Z, Wang J, et al. Cardiac resynchronization therapy via left bundle branch pacing vs. optimized biventricular pacing with adaptive algorithm in heart failure with left bundle branch block: a prospective, multi-centre, observational study. Europace. 2022;24(5):807–16. 10.1093/europace/euab249 PMC9071084 34718539

[36] Žižek D, Antolič B, Mežnar AZ, Zavrl-Džananović D, Jan M, Štublar J, et al. Biventricular versus His bundle pacing after atrioventricular node ablation in heart failure patients with narrow QRS. Acta Cardiologica. 2022;77(3):222–30. 10.1080/00015385.2021.1903196 34078244

[37] Hua J, Chen Y, Yu J, Xiong Q, Xia Z, Xia Z (2022). Long-term outcomes of left bundle branch area pacing versus biventricular pacing in patients with heart failure and complete left bundle branch block. Heart Vessels.

[38] Vijayaraman P, Zalavadia D, Haseeb A, Dye C, Madan N, Skeete JR (2022). Clinical outcomes of conduction system pacing compared to biventricular pacing in patients requiring cardiac resynchronization therapy. Heart Rhythm.

[39] Poole JE, Singh JP, Birgersdotter-Green U (2016). QRS duration or QRS morphology: what really matters in cardiac resynchronization therapy?. J Am Coll Cardiol.

[40] Sutton MG, Plappert T, Hilpisch KE, Abraham WT, Hayes DL, Chinchoy E (2006). Sustained reverse left ventricular structural remodeling with cardiac resynchronization at one year is a function of etiology: quantitative Doppler echocardiographic evidence from the Multicenter InSync Randomized Clinical Evaluation (MIRACLE). Circulation.

[41] Padala SK, Cabrera JA, Ellenbogen KA (2021). Anatomy of the cardiac conduction system. Pacing Clin Electrophysiol.

[42] Huang W, Su L, Wu S, Xu L, Xiao F, Zhou X, et al. Benefits of permanent his bundle pacing combined with atrioventricular node ablation in atrial fibrillation patients with heart failure with both preserved and reduced left ventricular ejection fraction. J Am Heart Assoc. 2017;6(4):e005309. 10.1161/JAHA.116.005309 PMC5533020 28365568

[43] Ploux S, Eschalier R, Whinnett ZI, Lumens J, Derval N, Sacher F (2015). Electrical dyssynchrony induced by biventricular pacing: implications for patient selection and therapy improvement. Heart Rhythm.

