# Supplementary material for: His-Purkinje system pacing versus biventricular pacing in clinical efficacy: a systematic review and meta-analysis
Source: BMC Cardiovasc Disord. 2023 Jun 3;23:285. doi: 10.1186/s12872-023-03307-7 (PMC10239598; doi:10.1186/s12872-023-03307-7)
Supplement: Supplementary file 1 — Additional file 1. [file 12872_2023_3307_MOESM1_ESM.docx]

**On-Line Only Data Supplement**

**His-Purkinje System Pacing versus Biventricular Pacing in clinical efficacy: A Systematic Review and Meta-Analysis**

**Ya Wang^1^, Fangchao Liu^1^, Mengyao Liu^1^, Zefeng Wang^2^, Xiangfeng Lu^1^, Jianfeng Huang^1^, Dongfeng Gu^1 3*^**

1 Department of Epidemiology, Fuwai Hospital, State Key Laboratory of Cardiovascular Disease, National Center for Cardiovascular Diseases, Chinese Academy of Medical Sciences and Peking Union Medical College, Beijing, China

2 Department of Cardiology, Beijing Anzhen Hospital Affiliated to Capital Medical University, Beijing, China.

3 School of Medicine, Southern University of Science and Technology, Shenzhen, 518055, China.

**Corresponding Author**

Dongfeng Gu, MD, PhD

Email: [gudongfeng@cashq.ac.cn](mailto:gudongfeng@cashq.ac.cn)

Address: No. 167 Beilishi Road, Beijing, 100037, China.

Tel: +861068331752, China

ORCID: 0000-0002-2781-7825

**PubMed Search strategy**

#1: (left bundle branch area pacing) AND (biventricular) OR (cardiac resynchronization therapy) OR (CRT);

#2: (His-bundle pacing) AND (biventricular)) OR (cardiac resynchronization therapy) OR (CRT);

#3: (conduction system pacing) AND (biventricular)) OR (cardiac resynchronization therapy) OR (CRT);

#4: (#1 AND #2 AND #3) Filters: Humans；

#5: (#1 AND #2 AND #3) Filters: (Clinical Trial) OR (Randomized Controlled Trial)

**EMBASE** **Search strategy**

#1: 'left bundle branch area pacing':ti,ab,kw AND biventricular:ti,ab,kw OR 'cardiac resynchronization therapy':ti,ab,kw OR crt:ti,ab,kw；

#2: 'his-bundle pacing':ti,ab,kw AND biventricular:ti,ab,kw OR 'cardiac resynchronization therapy':ti,ab,kw OR crt:ti,ab,kw；

#3: ' conduction system pacing ':ti,ab,kw AND biventricular:ti,ab,kw OR 'cardiac resynchronization therapy':ti,ab,kw OR crt:ti,ab,kw；

#4: (#1 AND #2 AND #3) Filters: [humans]/lim AND [clinical study];

#5: (#1 AND #2 AND #3) Filters: [controlled clinical trial]/lim OR [randomized controlled trial]/lim

**Cochrane’s Library Search strategy**

#1: “left bundle branch area pacing” [ti, ab, kw] and “biventricular” [ti, ab, kw] or “cardiac resynchronization therapy” [ti, ab, kw] or “CRT” [ti, ab, kw];

#2: “his-bundle pacing” [ti, ab, kw] and “biventricular” [ti, ab, kw] or “cardiac resynchronization therapy” [ti, ab, kw] or “CRT” [ti, ab, kw];

#3: “conduction system pacing” [ti, ab, kw] and “biventricular” [ti, ab, kw] or “cardiac resynchronization therapy” [ti, ab, kw] or “CRT” [ti, ab, kw];

#4: (#1 AND #2 AND #3) Filters: [trials]/lim(Word variations have been searched);

**Web of Science Search strategy**

#1: (((TS = (left bundle branch area pacing)) AND TS=(biventricular)) OR TS = (cardiac resynchronization therapy)) OR TS=(CRT);

#2: (((TS = (his-bundle pacing)) AND TS=(biventricular)) OR TS = (cardiac resynchronization therapy)) OR TS=(CRT);

#3: (((TS = (his-bundle pacing)) AND TS=(biventricular)) OR TS = (cardiac resynchronization therapy)) OR TS=(CRT);

#4: (#1 AND #2 AND #3) Filters: [Clinical Trials]/Ref;

#5: (#1 AND #2 AND #3) Filters: [Humans]/Ref;


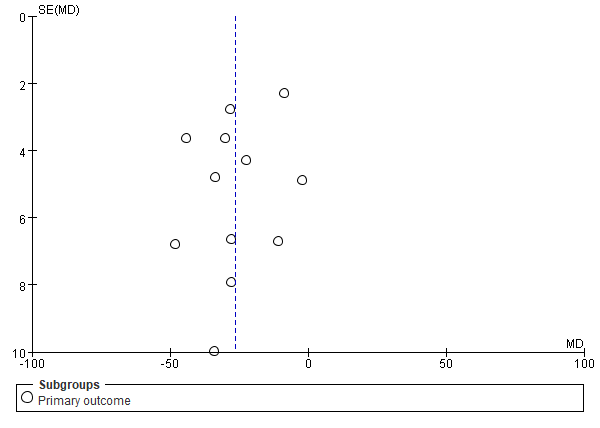
A B


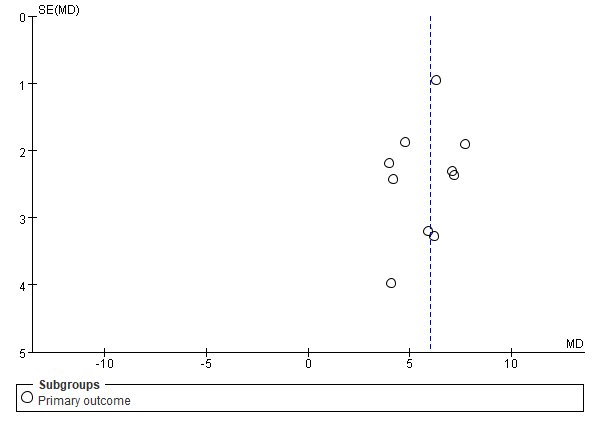


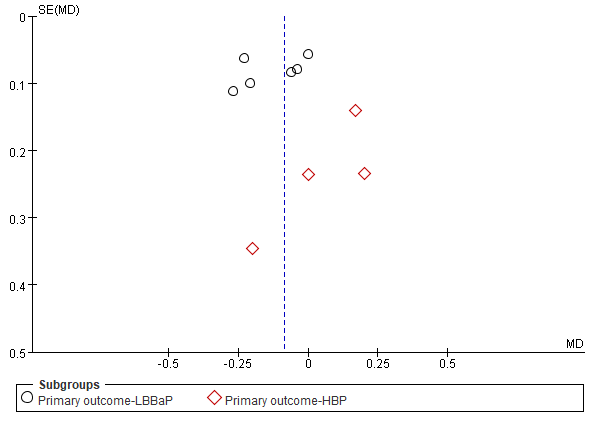
C D


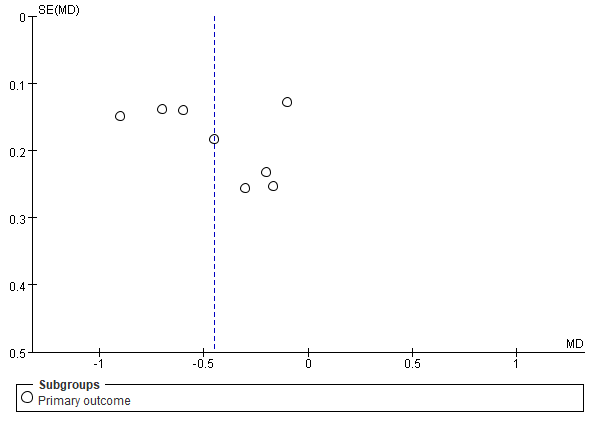


Figure S1. Funnel plots for the meta-analysis on the association of pacing implantation intervention with QRSd decreasing (A); LVEF improvement (B); NYHA improvement (C) and Threshold change (D); SE, standard error.
